# 2-[(4-Bromo­phen­yl)imino­meth­yl]-3,5-dimethoxy­phenol

**DOI:** 10.1107/S1600536809011416

**Published:** 2009-03-31

**Authors:** Işın Kılıç, Erbil Ağar, Ferda Erşahin, Şamil Işık

**Affiliations:** a Department of Physics, Faculty of Arts & Science, Ondokuz Mayıs University, TR-55139 Kurupelit-Samsun, Turkey; b Department of Chemistry, Faculty of Arts & Science, Ondokuz Mayıs University, 55139 Samsun, Turkey

## Abstract

There are two independent mol­ecules in the asymmetric unit of the title compound, C_15_H_14_BrNO_3_, with very similar geometrical parameters. Each mol­ecule adopts the phenol–imine tautomeric form, with strong intra­molecular O—H⋯N hydrogen bonds. The two mol­ecules are non-planar, the dihedral angles between the two aromatic rings being are 24.6 (2) and 30.30 (13)°.

## Related literature

For bond-length data, see: Petek *et al.* (2007 ▶).
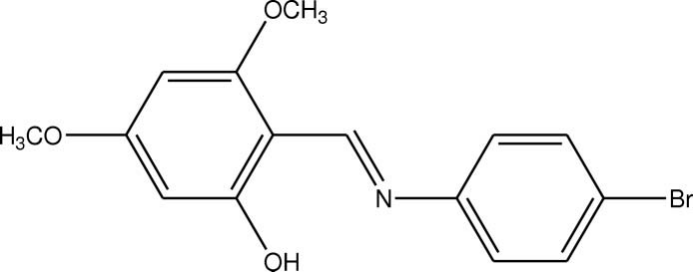


## Experimental

### 

#### Crystal data

C_15_H_14_BrNO_3_
*M* *_r_* = 336.18
Triclinic, 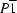

*a* = 8.2655 (5) Å
*b* = 9.7305 (6) Å
*c* = 18.3806 (11) Å
α = 97.177 (5)°
β = 92.796 (5)°
γ = 106.214 (5)°
*V* = 1402.94 (15) Å^3^
*Z* = 4
Mo *K*α radiation
μ = 2.94 mm^−1^
*T* = 296 K
0.67 × 0.38 × 0.09 mm

#### Data collection

Stoe IPDS-2 diffractometer
Absorption correction: integration (*X-RED*; Stoe & Cie, 2002 ▶) *T*_min_ = 0.421, *T*_max_ = 0.839
20096 measured reflections
5514 independent reflections
3901 reflections with *I* > 2σ(*I*)
*R*_int_ = 0.080

#### Refinement

*R*[*F* ^2^ > 2σ(*F* ^2^)] = 0.050
*wR*(*F* ^2^) = 0.112
*S* = 1.02
5514 reflections
369 parameters
H atoms treated by a mixture of independent and constrained refinement
Δρ_max_ = 0.61 e Å^−3^
Δρ_min_ = −0.92 e Å^−3^

### 

Data collection: *X-AREA* (Stoe & Cie, 2002 ▶); cell refinement: *X-AREA*; data reduction: *X-RED32* (Stoe & Cie, 2002 ▶); program(s) used to solve structure: *SHELXS97* (Sheldrick, 2008 ▶); program(s) used to refine structure: *SHELXL97* (Sheldrick, 2008 ▶); molecular graphics: *ORTEP-3 for Windows* (Farrugia, 1997 ▶); software used to prepare material for publication: *WinGX* (Farrugia, 1999 ▶).

## Supplementary Material

Crystal structure: contains datablocks I, global. DOI: 10.1107/S1600536809011416/bt2917sup1.cif

Structure factors: contains datablocks I. DOI: 10.1107/S1600536809011416/bt2917Isup2.hkl

Additional supplementary materials:  crystallographic information; 3D view; checkCIF report

## Figures and Tables

**Table 1:** Hydrogen-bond geometry (Å, °)

*D*—H⋯*A*	*D*—H	H⋯*A*	*D*⋯*A*	*D*—H⋯*A*
O1—H1⋯N1	0.97 (5)	1.69 (5)	2.564 (4)	149 (5)
O4—H4⋯N2	0.83 (5)	1.80 (5)	2.564 (4)	150 (5)

**Fig. 1.**
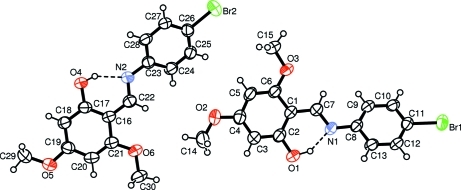
The molecular structure of the title compound, showing the atom-numbering scheme. Displacement ellipsoids are at the 50% probability level Dashed lines indicate intramolecular hydrogen bond.

**Fig. 2.**
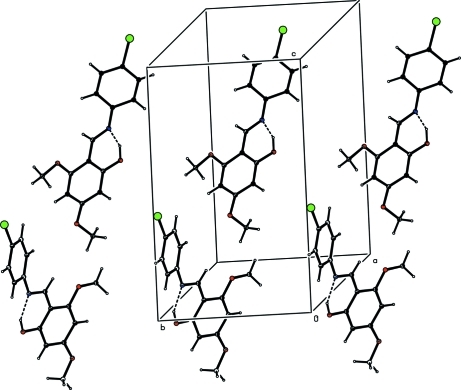
The crystal packing of the title compound. Dashed lines indicate intramolecular hydrogen bond.

## supplementary crystallographic information

### Comment

The extensive application of Schiff bases in industry and in analytical determinations has attracted attention for decades. The overall behaviour of these compounds has been ascribed to a proton-transfer reaction between a phenol-imine and a keto-amine tautomer. It is claimed that phenol-imine tautomerism is dominant in salicylaldimine, while the keto-amine form is preferred in naphthaldimine Schiff bases, depending on the solvent polarities. Our X-ray investigation of the title compound  has indicated that the phenol-imine tautomer is favoured over the keto-amine tautomer.

An *ORTEP* view of the molecule   is shown in Fig. 1. There are two independent molecules in the asymmetric unit which have very similar geometrical parameters. Both molecules adopt the phenol-imine tautomeric form and have a strong intramolecular O—H···N hydrogen bond whose details are given in Table 1. The C7—N1 [1.296 (4) Å] and C22—N2 [1.296 (4) Å] bond distances are of double-bond character, whereas, the C2—O1 [1.344 (4) Å] and C17—O4 [1.342 (4) Å] distances are single bonds. These distances are similar to other values reported in the literature [1.2889 (15) and 1.2891 (14) Å for C=N and 1.3486 (16) and 1.3443 (15) Å for C—O, respectively; Petek *et al.* (2007)]. Both molecules are not planar; the dihedral angle between the aromatic rings are 24.6 (2) and 30.30 (13) °, respectively.

### Experimental

2-(4-Bromophenylimino)methyl-3,5-dimethoxyphenol was prepared by reflux a mixture of a solution containing 2-hydroxy-4, 6-dimethoxybenzaldehyde (0.02 g 0.11 mmol) in 20 ml ethanol and a solution containing 4-bromoaniline (0.019 g 0.11 mmol) in 20 ml ethanol. The reaction mixture was stirred for 1 hunder reflux. Crystals of 2-(4-Bromophenylimino)methyl-3,5-dimethoxyphenol suitable for X-ray analysis were obtained from ethylalcohol by slow evaporation (yield % 69; m.p.380–382 K).

### Refinement

All H atoms bonded to C were positioned geometrically and treated using a riding model, fixing the bond lengths at 0.93 and 0.96 Å for C_amoatic_-H or C_methyl_H, respectively. The displacement parameters of the H atoms were constrained as *U*_iso_(H) = 1.2*U*_eq_(C_aromatic_) or 1.5*U*_eq_(C_methyl_). The positions of the hydroxyl H atoms were obtained from an electron density difference map   and were refined freely.

### Crystal data

**Table:**

C_15_H_14_BrNO_3_	*Z* = 4
*M**_r_* = 336.18	*F*(000) = 680
Triclinic, *P*1	*D*_x_ = 1.592 Mg m^−^^3^
Hall symbol: -P 1	Mo *K*α radiation, λ = 0.71073 Å
*a* = 8.2655 (5) Å	Cell parameters from 26370 reflections
*b* = 9.7305 (6) Å	θ = 2.2–29.8°
*c* = 18.3806 (11) Å	µ = 2.94 mm^−^^1^
α = 97.177 (5)°	*T* = 296 K
β = 92.796 (5)°	Plate, yellow
γ = 106.214 (5)°	0.67 × 0.38 × 0.09 mm
*V* = 1402.94 (15) Å^3^	

### Data collection

**Table:**

Stoe IPDS-2 diffractometer	5514 independent reflections
Radiation source: fine-focus sealed tube	3901 reflections with *I* > 2σ(*I*)
plane graphite	*R*_int_ = 0.080
Detector resolution: 6.67 pixels mm^-1^	θ_max_ = 26.0°, θ_min_ = 2.2°
rotation method scans	*h* = −10→10
Absorption correction: integration (*X-RED*; Stoe & Cie, 2002)	*k* = −12→12
*T*_min_ = 0.421, *T*_max_ = 0.839	*l* = −22→22
20096 measured reflections	

### Refinement

**Table:**

Refinement on *F*^2^	Primary atom site location: structure-invariant direct methods
Least-squares matrix: full	Secondary atom site location: difference Fourier map
*R*[*F*^2^ > 2σ(*F*^2^)] = 0.050	Hydrogen site location: inferred from neighbouring sites
*wR*(*F*^2^) = 0.112	H atoms treated by a mixture of independent and constrained refinement
*S* = 1.02	*w* = 1/[σ^2^(*F*_o_^2^) + (0.0586*P*)^2^] where *P* = (*F*_o_^2^ + 2*F*_c_^2^)/3
5514 reflections	(Δ/σ)_max_ = 0.001
369 parameters	Δρ_max_ = 0.61 e Å^−^^3^
0 restraints	Δρ_min_ = −0.92 e Å^−^^3^

### Special details

**Table:**

Experimental. 360 frames, detector distance = 100 mm
Geometry. All e.s.d.'s (except the e.s.d. in the dihedral angle between two l.s. planes) are estimated using the full covariance matrix. The cell e.s.d.'s are taken into account individually in the estimation of e.s.d.'s in distances, angles and torsion angles; correlations between e.s.d.'s in cell parameters are only used when they are defined by crystal symmetry. An approximate (isotropic) treatment of cell e.s.d.'s is used for estimating e.s.d.'s involving l.s. planes.
Refinement. Refinement of *F*^2^ against ALL reflections. The weighted *R*-factor *wR* and goodness of fit *S* are based on *F*^2^, conventional *R*-factors *R* are based on *F*, with *F* set to zero for negative *F*^2^. The threshold expression of *F*^2^ > σ(*F*^2^) is used only for calculating *R*-factors(gt) *etc*. and is not relevant to the choice of reflections for refinement. *R*-factors based on *F*^2^ are statistically about twice as large as those based on *F*, and *R*- factors based on ALL data will be even larger.

### Fractional atomic coordinates and isotropic or equivalent isotropic displacement parameters (Å^2^)

**Table:**

	*x*	*y*	*z*	*U*_iso_*/*U*_eq_	
H1	0.862 (6)	0.544 (5)	0.509 (3)	0.086 (15)*	
H4	0.128 (6)	0.946 (5)	0.005 (3)	0.079 (15)*	
C1	0.6535 (4)	0.6668 (4)	0.50157 (19)	0.0470 (8)	
C2	0.7277 (5)	0.6064 (4)	0.4429 (2)	0.0542 (9)	
C3	0.6849 (5)	0.6161 (5)	0.3700 (2)	0.0581 (10)	
H3	0.7331	0.5735	0.3321	0.070*	
C4	0.5703 (5)	0.6897 (4)	0.3547 (2)	0.0555 (9)	
C5	0.4951 (5)	0.7517 (4)	0.4110 (2)	0.0581 (10)	
H5	0.4181	0.8014	0.3999	0.070*	
C6	0.5340 (5)	0.7397 (4)	0.4822 (2)	0.0525 (9)	
C7	0.6914 (4)	0.6516 (4)	0.5757 (2)	0.0486 (8)	
H7	0.6351	0.6885	0.6124	0.058*	
C8	0.8394 (4)	0.5718 (4)	0.66721 (19)	0.0460 (8)	
C9	0.8251 (4)	0.6672 (4)	0.7279 (2)	0.0506 (9)	
H9	0.7870	0.7464	0.7210	0.061*	
C10	0.8666 (5)	0.6459 (4)	0.7981 (2)	0.0503 (9)	
H10	0.8573	0.7103	0.8384	0.060*	
C11	0.9221 (4)	0.5282 (4)	0.80813 (19)	0.0480 (8)	
C12	0.9410 (4)	0.4343 (4)	0.7486 (2)	0.0499 (9)	
H12	0.9809	0.3562	0.7558	0.060*	
C13	0.9008 (5)	0.4565 (4)	0.6792 (2)	0.0522 (9)	
H13	0.9145	0.3936	0.6391	0.063*	
C14	0.5657 (6)	0.6265 (6)	0.2249 (2)	0.0751 (12)	
H14A	0.5251	0.6514	0.1800	0.113*	
H14B	0.5140	0.5257	0.2269	0.113*	
H14C	0.6863	0.6451	0.2264	0.113*	
C15	0.3157 (6)	0.8384 (6)	0.5262 (3)	0.0840 (16)	
H15A	0.2800	0.8751	0.5716	0.126*	
H15B	0.2274	0.7562	0.5021	0.126*	
H15C	0.3394	0.9123	0.4949	0.126*	
C16	0.3436 (4)	0.8322 (4)	−0.00258 (19)	0.0472 (8)	
C17	0.2508 (5)	0.8723 (4)	−0.0580 (2)	0.0493 (9)	
C18	0.2770 (5)	0.8456 (4)	−0.1320 (2)	0.0534 (9)	
H18	0.2147	0.8736	−0.1679	0.064*	
C19	0.3969 (5)	0.7772 (4)	−0.1511 (2)	0.0525 (9)	
C20	0.4941 (5)	0.7381 (4)	−0.0976 (2)	0.0567 (10)	
H20	0.5761	0.6936	−0.1114	0.068*	
C21	0.4692 (5)	0.7651 (4)	−0.0252 (2)	0.0506 (9)	
C22	0.3103 (5)	0.8518 (4)	0.0720 (2)	0.0495 (9)	
H22	0.3742	0.8228	0.1069	0.059*	
C23	0.1593 (5)	0.9256 (4)	0.16780 (19)	0.0485 (8)	
C24	0.2798 (5)	0.9472 (4)	0.2266 (2)	0.0543 (9)	
H24	0.3905	0.9508	0.2176	0.065*	
C25	0.2378 (5)	0.9636 (4)	0.2982 (2)	0.0539 (9)	
H25	0.3197	0.9792	0.3372	0.065*	
C26	0.0739 (5)	0.9566 (4)	0.31126 (19)	0.0511 (9)	
C27	−0.0479 (5)	0.9373 (4)	0.2532 (2)	0.0528 (9)	
H27	−0.1583	0.9347	0.2624	0.063*	
C28	−0.0043 (5)	0.9220 (4)	0.1826 (2)	0.0523 (9)	
H28	−0.0860	0.9089	0.1438	0.063*	
C29	0.3267 (6)	0.7663 (5)	−0.2796 (2)	0.0694 (12)	
H29A	0.3624	0.7342	−0.3260	0.104*	
H29B	0.3382	0.8680	−0.2755	0.104*	
H29C	0.2106	0.7143	−0.2766	0.104*	
C30	0.6916 (6)	0.6705 (6)	0.0127 (3)	0.0811 (15)	
H30A	0.7415	0.6483	0.0563	0.122*	
H30B	0.7753	0.7407	−0.0083	0.122*	
H30C	0.6499	0.5841	−0.0223	0.122*	
Br1	0.97872 (6)	0.49642 (5)	0.90475 (2)	0.06820 (16)	
Br2	0.01410 (7)	0.97819 (6)	0.40896 (2)	0.07682 (18)	
N1	0.8025 (4)	0.5876 (3)	0.59359 (16)	0.0525 (8)	
N2	0.1932 (4)	0.9090 (3)	0.09355 (16)	0.0516 (7)	
O1	0.8425 (4)	0.5365 (4)	0.45644 (17)	0.0749 (9)	
O2	0.5240 (4)	0.7113 (3)	0.28614 (15)	0.0706 (8)	
O3	0.4661 (4)	0.7956 (4)	0.54100 (15)	0.0725 (9)	
O4	0.1272 (4)	0.9326 (3)	−0.04128 (16)	0.0646 (8)	
O5	0.4290 (4)	0.7403 (3)	−0.22137 (15)	0.0656 (7)	
O6	0.5547 (3)	0.7280 (3)	0.03109 (15)	0.0675 (8)	

### Atomic displacement parameters (Å^2^)

**Table:**

	*U*^11^	*U*^22^	*U*^33^	*U*^12^	*U*^13^	*U*^23^
C1	0.0424 (19)	0.048 (2)	0.053 (2)	0.0165 (18)	−0.0015 (15)	0.0110 (16)
C2	0.052 (2)	0.057 (2)	0.059 (2)	0.022 (2)	0.0009 (17)	0.0162 (18)
C3	0.057 (2)	0.070 (3)	0.054 (2)	0.028 (2)	0.0059 (17)	0.0131 (19)
C4	0.052 (2)	0.062 (2)	0.052 (2)	0.014 (2)	−0.0013 (17)	0.0146 (18)
C5	0.058 (2)	0.063 (2)	0.060 (2)	0.029 (2)	−0.0084 (18)	0.0120 (19)
C6	0.051 (2)	0.056 (2)	0.055 (2)	0.025 (2)	−0.0042 (17)	0.0076 (18)
C7	0.045 (2)	0.051 (2)	0.0513 (19)	0.0163 (18)	−0.0007 (15)	0.0080 (16)
C8	0.0403 (19)	0.049 (2)	0.0529 (19)	0.0173 (17)	0.0007 (15)	0.0138 (16)
C9	0.049 (2)	0.050 (2)	0.060 (2)	0.0242 (19)	0.0024 (16)	0.0118 (17)
C10	0.051 (2)	0.049 (2)	0.056 (2)	0.0240 (19)	0.0046 (16)	0.0070 (17)
C11	0.0424 (19)	0.052 (2)	0.0510 (19)	0.0137 (18)	0.0001 (15)	0.0143 (17)
C12	0.048 (2)	0.049 (2)	0.060 (2)	0.0255 (18)	−0.0012 (16)	0.0111 (17)
C13	0.051 (2)	0.053 (2)	0.058 (2)	0.0256 (19)	−0.0006 (17)	0.0040 (17)
C14	0.080 (3)	0.094 (3)	0.050 (2)	0.025 (3)	−0.004 (2)	0.010 (2)
C15	0.088 (3)	0.111 (4)	0.079 (3)	0.070 (3)	0.005 (3)	0.016 (3)
C16	0.0422 (19)	0.046 (2)	0.056 (2)	0.0186 (18)	0.0043 (16)	0.0053 (16)
C17	0.048 (2)	0.050 (2)	0.055 (2)	0.0199 (19)	0.0128 (16)	0.0100 (17)
C18	0.051 (2)	0.062 (2)	0.055 (2)	0.025 (2)	0.0101 (17)	0.0151 (18)
C19	0.051 (2)	0.056 (2)	0.053 (2)	0.0180 (19)	0.0119 (17)	0.0084 (17)
C20	0.046 (2)	0.063 (2)	0.067 (2)	0.026 (2)	0.0131 (18)	0.0062 (19)
C21	0.045 (2)	0.051 (2)	0.058 (2)	0.0183 (19)	0.0055 (16)	0.0055 (17)
C22	0.049 (2)	0.044 (2)	0.057 (2)	0.0160 (18)	0.0039 (17)	0.0079 (16)
C23	0.058 (2)	0.0421 (19)	0.0480 (19)	0.0199 (19)	0.0047 (16)	0.0058 (15)
C24	0.048 (2)	0.058 (2)	0.059 (2)	0.020 (2)	0.0014 (17)	0.0056 (18)
C25	0.059 (2)	0.058 (2)	0.048 (2)	0.024 (2)	−0.0044 (17)	0.0058 (17)
C26	0.070 (3)	0.045 (2)	0.0426 (18)	0.025 (2)	0.0040 (17)	0.0030 (15)
C27	0.052 (2)	0.062 (2)	0.051 (2)	0.031 (2)	0.0032 (16)	0.0036 (17)
C28	0.052 (2)	0.056 (2)	0.056 (2)	0.030 (2)	−0.0013 (17)	0.0026 (17)
C29	0.071 (3)	0.092 (3)	0.050 (2)	0.032 (3)	0.0124 (19)	0.006 (2)
C30	0.073 (3)	0.104 (4)	0.084 (3)	0.059 (3)	−0.002 (2)	0.002 (3)
Br1	0.0863 (3)	0.0739 (3)	0.0528 (2)	0.0335 (3)	−0.0004 (2)	0.0194 (2)
Br2	0.0908 (4)	0.0951 (4)	0.0472 (2)	0.0325 (3)	0.0114 (2)	0.0046 (2)
N1	0.0512 (18)	0.0610 (19)	0.0530 (17)	0.0257 (16)	0.0020 (14)	0.0166 (15)
N2	0.0568 (19)	0.0546 (18)	0.0509 (17)	0.0273 (17)	0.0082 (14)	0.0085 (14)
O1	0.081 (2)	0.106 (2)	0.0632 (18)	0.066 (2)	0.0106 (15)	0.0200 (17)
O2	0.078 (2)	0.091 (2)	0.0512 (15)	0.0359 (18)	−0.0052 (13)	0.0183 (15)
O3	0.0772 (19)	0.103 (2)	0.0572 (16)	0.0624 (19)	−0.0024 (14)	0.0064 (15)
O4	0.0738 (19)	0.088 (2)	0.0530 (16)	0.0548 (18)	0.0112 (14)	0.0130 (15)
O5	0.0667 (17)	0.085 (2)	0.0554 (16)	0.0364 (17)	0.0178 (13)	0.0085 (14)
O6	0.0665 (18)	0.088 (2)	0.0644 (17)	0.0503 (17)	0.0023 (13)	0.0079 (15)

### Geometric parameters (Å, °)

**Table:**

C1—C2	1.412 (5)	C16—C22	1.412 (5)
C1—C7	1.418 (5)	C16—C21	1.425 (5)
C1—C6	1.424 (4)	C17—O4	1.342 (4)
C2—O1	1.344 (4)	C17—C18	1.392 (5)
C2—C3	1.390 (5)	C18—C19	1.376 (5)
C3—C4	1.375 (5)	C18—H18	0.9300
C3—H3	0.9300	C19—O5	1.356 (4)
C4—O2	1.358 (4)	C19—C20	1.394 (5)
C4—C5	1.392 (5)	C20—C21	1.361 (5)
C5—C6	1.358 (5)	C20—H20	0.9300
C5—H5	0.9300	C21—O6	1.366 (4)
C6—O3	1.365 (4)	C22—N2	1.299 (4)
C7—N1	1.296 (4)	C22—H22	0.9300
C7—H7	0.9300	C23—C28	1.384 (5)
C8—C13	1.390 (4)	C23—C24	1.387 (5)
C8—C9	1.392 (5)	C23—N2	1.405 (4)
C8—N1	1.409 (4)	C24—C25	1.379 (5)
C9—C10	1.373 (5)	C24—H24	0.9300
C9—H9	0.9300	C25—C26	1.372 (5)
C10—C11	1.376 (5)	C25—H25	0.9300
C10—H10	0.9300	C26—C27	1.387 (5)
C11—C12	1.380 (5)	C26—Br2	1.888 (3)
C11—Br1	1.899 (3)	C27—C28	1.365 (5)
C12—C13	1.362 (5)	C27—H27	0.9300
C12—H12	0.9300	C28—H28	0.9300
C13—H13	0.9300	C29—O5	1.422 (5)
C14—O2	1.426 (5)	C29—H29A	0.9600
C14—H14A	0.9600	C29—H29B	0.9600
C14—H14B	0.9600	C29—H29C	0.9600
C14—H14C	0.9600	C30—O6	1.432 (4)
C15—O3	1.441 (4)	C30—H30A	0.9600
C15—H15A	0.9600	C30—H30B	0.9600
C15—H15B	0.9600	C30—H30C	0.9600
C15—H15C	0.9600	O1—H1	0.97 (5)
C16—C17	1.403 (5)	O4—H4	0.83 (5)
			
C2—C1—C7	121.9 (3)	O4—C17—C16	120.5 (3)
C2—C1—C6	116.4 (3)	C18—C17—C16	121.9 (3)
C7—C1—C6	121.7 (3)	C19—C18—C17	118.8 (3)
O1—C2—C3	118.0 (3)	C19—C18—H18	120.6
O1—C2—C1	120.2 (3)	C17—C18—H18	120.6
C3—C2—C1	121.8 (3)	O5—C19—C18	124.2 (3)
C4—C3—C2	119.2 (4)	O5—C19—C20	114.7 (3)
C4—C3—H3	120.4	C18—C19—C20	121.1 (3)
C2—C3—H3	120.4	C21—C20—C19	120.1 (3)
O2—C4—C3	124.5 (4)	C21—C20—H20	120.0
O2—C4—C5	114.7 (3)	C19—C20—H20	120.0
C3—C4—C5	120.8 (3)	C20—C21—O6	124.6 (3)
C6—C5—C4	120.2 (3)	C20—C21—C16	121.1 (3)
C6—C5—H5	119.9	O6—C21—C16	114.3 (3)
C4—C5—H5	119.9	N2—C22—C16	122.1 (3)
C5—C6—O3	124.5 (3)	N2—C22—H22	119.0
C5—C6—C1	121.6 (3)	C16—C22—H22	119.0
O3—C6—C1	113.9 (3)	C28—C23—C24	118.5 (3)
N1—C7—C1	121.7 (3)	C28—C23—N2	117.5 (3)
N1—C7—H7	119.1	C24—C23—N2	124.1 (3)
C1—C7—H7	119.1	C25—C24—C23	121.0 (3)
C13—C8—C9	118.4 (3)	C25—C24—H24	119.5
C13—C8—N1	117.3 (3)	C23—C24—H24	119.5
C9—C8—N1	124.2 (3)	C26—C25—C24	119.3 (3)
C10—C9—C8	120.7 (3)	C26—C25—H25	120.3
C10—C9—H9	119.6	C24—C25—H25	120.3
C8—C9—H9	119.6	C25—C26—C27	120.6 (3)
C9—C10—C11	119.4 (3)	C25—C26—Br2	119.9 (3)
C9—C10—H10	120.3	C27—C26—Br2	119.6 (3)
C11—C10—H10	120.3	C28—C27—C26	119.5 (3)
C10—C11—C12	120.7 (3)	C28—C27—H27	120.3
C10—C11—Br1	119.9 (3)	C26—C27—H27	120.3
C12—C11—Br1	119.3 (2)	C27—C28—C23	121.2 (3)
C13—C12—C11	119.6 (3)	C27—C28—H28	119.4
C13—C12—H12	120.2	C23—C28—H28	119.4
C11—C12—H12	120.2	O5—C29—H29A	109.5
C12—C13—C8	121.0 (3)	O5—C29—H29B	109.5
C12—C13—H13	119.5	H29A—C29—H29B	109.5
C8—C13—H13	119.5	O5—C29—H29C	109.5
O2—C14—H14A	109.5	H29A—C29—H29C	109.5
O2—C14—H14B	109.5	H29B—C29—H29C	109.5
H14A—C14—H14B	109.5	O6—C30—H30A	109.5
O2—C14—H14C	109.5	O6—C30—H30B	109.5
H14A—C14—H14C	109.5	H30A—C30—H30B	109.5
H14B—C14—H14C	109.5	O6—C30—H30C	109.5
O3—C15—H15A	109.5	H30A—C30—H30C	109.5
O3—C15—H15B	109.5	H30B—C30—H30C	109.5
H15A—C15—H15B	109.5	C7—N1—C8	121.7 (3)
O3—C15—H15C	109.5	C22—N2—C23	121.5 (3)
H15A—C15—H15C	109.5	C2—O1—H1	108 (3)
H15B—C15—H15C	109.5	C4—O2—C14	117.9 (3)
C17—C16—C22	121.9 (3)	C6—O3—C15	117.3 (3)
C17—C16—C21	117.0 (3)	C17—O4—H4	108 (3)
C22—C16—C21	121.1 (3)	C19—O5—C29	118.4 (3)
O4—C17—C18	117.5 (3)	C21—O6—C30	117.0 (3)
			
C7—C1—C2—O1	−2.5 (6)	C17—C18—C19—C20	−1.6 (6)
C6—C1—C2—O1	179.6 (4)	O5—C19—C20—C21	−177.8 (4)
C7—C1—C2—C3	177.2 (4)	C18—C19—C20—C21	1.2 (6)
C6—C1—C2—C3	−0.7 (6)	C19—C20—C21—O6	178.3 (4)
O1—C2—C3—C4	−178.7 (4)	C19—C20—C21—C16	0.5 (6)
C1—C2—C3—C4	1.6 (6)	C17—C16—C21—C20	−1.6 (6)
C2—C3—C4—O2	177.2 (4)	C22—C16—C21—C20	175.9 (4)
C2—C3—C4—C5	−1.2 (6)	C17—C16—C21—O6	−179.7 (3)
O2—C4—C5—C6	−178.7 (4)	C22—C16—C21—O6	−2.2 (5)
C3—C4—C5—C6	−0.2 (7)	C17—C16—C22—N2	−0.7 (6)
C4—C5—C6—O3	−179.4 (4)	C21—C16—C22—N2	−178.0 (4)
C4—C5—C6—C1	1.2 (7)	C28—C23—C24—C25	0.6 (6)
C2—C1—C6—C5	−0.7 (6)	N2—C23—C24—C25	179.6 (3)
C7—C1—C6—C5	−178.6 (4)	C23—C24—C25—C26	0.7 (6)
C2—C1—C6—O3	179.8 (4)	C24—C25—C26—C27	−1.7 (6)
C7—C1—C6—O3	1.9 (6)	C24—C25—C26—Br2	179.8 (3)
C2—C1—C7—N1	3.5 (6)	C25—C26—C27—C28	1.4 (6)
C6—C1—C7—N1	−178.7 (4)	Br2—C26—C27—C28	179.9 (3)
C13—C8—C9—C10	−1.6 (6)	C26—C27—C28—C23	0.0 (6)
N1—C8—C9—C10	−178.8 (4)	C24—C23—C28—C27	−0.9 (6)
C8—C9—C10—C11	−0.3 (6)	N2—C23—C28—C27	−180.0 (3)
C9—C10—C11—C12	1.9 (6)	C1—C7—N1—C8	−179.6 (4)
C9—C10—C11—Br1	−179.6 (3)	C13—C8—N1—C7	153.9 (4)
C10—C11—C12—C13	−1.4 (6)	C9—C8—N1—C7	−28.9 (6)
Br1—C11—C12—C13	−179.9 (3)	C16—C22—N2—C23	178.9 (4)
C11—C12—C13—C8	−0.6 (6)	C28—C23—N2—C22	−150.7 (4)
C9—C8—C13—C12	2.1 (6)	C24—C23—N2—C22	30.3 (6)
N1—C8—C13—C12	179.4 (4)	C3—C4—O2—C14	14.8 (6)
C22—C16—C17—O4	0.7 (6)	C5—C4—O2—C14	−166.7 (4)
C21—C16—C17—O4	178.2 (4)	C5—C6—O3—C15	15.8 (6)
C22—C16—C17—C18	−176.3 (4)	C1—C6—O3—C15	−164.8 (4)
C21—C16—C17—C18	1.2 (6)	C18—C19—O5—C29	−3.6 (6)
O4—C17—C18—C19	−176.7 (4)	C20—C19—O5—C29	175.3 (4)
C16—C17—C18—C19	0.4 (6)	C20—C21—O6—C30	6.8 (6)
C17—C18—C19—O5	177.2 (4)	C16—C21—O6—C30	−175.2 (4)

### Hydrogen-bond geometry (Å, °)

**Table:**

*D*—H···*A*	*D*—H	H···*A*	*D*···*A*	*D*—H···*A*
O1—H1···N1	0.97 (5)	1.69 (5)	2.564 (4)	149 (5)
O4—H4···N2	0.83 (5)	1.80 (5)	2.564 (4)	150 (5)
